# Membrane-based fractionation of red beetroot juice: bioactive compound enrichment and sugar removal

**DOI:** 10.1007/s10068-026-02213-w

**Published:** 2026-06-19

**Authors:** Aslı Arslan Kulcan

**Affiliations:** Department of Food Processing, Finike Vocational School, Akdeniz University, Antalya, Turkey

**Keywords:** Ultrafiltration, Diafiltration, Red beetroot juice, Betalains, Phenolic compounds

## Abstract

This study evaluated the effects of ultrafiltration (UF) and diafiltration (DF) on the chemical composition and selective fractionation of red beetroot juice. UF was performed using membranes with molecular weight cut-offs of 2 and 5 kDa to assess permeate flux, solute retention, and fouling behavior, with the 2 kDa membrane exhibiting higher rejection of phenolic compounds and betalains. DF was subsequently applied to the UF retentate obtained with the 2 kDa membrane to enhance the removal of low-molecular-weight sugars. The highest recovery values were observed at a diafiltration volume of 6, reaching 68.87% and 73.4% for phenolics and betalains, respectively. Under these conditions, 17.62% of the total soluble solids and 17.86% of the sugar content were retained in the retentate. Collectively, the integrated UF-DF process enabled the production of a concentrate enriched in phenolic compounds and betalains, while generating a permeate fraction enriched in sugars.

## Introduction

Red beetroot (*Beta vulgaris* L.) has gained increasing attention in recent years due to its high nutritional value and richness in bioactive compounds, particularly betalains and phenolic constituents. Betalains, which include the red-violet betacyanins and the yellow betaxanthins, are water-soluble pigments known for their strong antioxidant, anti-inflammatory, and potential health-promoting properties. In addition to pigments, red beetroot juice contains appreciable levels of phenolic compounds, sugars, and organic acids, all of which contribute to sensory attributes, functional quality, and nutritional value, making it a valuable ingredient in the food and beverage industry (Calva-Estrada et al., 2022; Prieto-Santiago et al., 2020; Wang et al., 2020). However, the presence of suspended solids, colloids, and high-molecular-weight compounds often poses technological challenges for processing and stabilization, necessitating the development of efficient clarification and concentration strategies. Betalains possess relatively low molecular weights (e.g., betaxanthin = 308 g/mol and betacyanin = 550 g/mol), yet their behavior during processing is strongly influenced by interactions with other juice components and processing conditions (Calva-Estrada et al., 2022).

In recent years, membrane separation technologies such as microfiltration (MF) and ultrafiltration (UF) have increasingly replaced conventional clarification techniques in fruit juice processing. Owing to their pressure-driven and highly selective nature, UF membranes enable the removal of colloidal particles, suspended solids, and high-molecular-weight compounds without the addition of chemicals and under mild thermal conditions. Membranes characterized by pore sizes of 1–50 nm or molecular weight cut-offs ranging from 1000 to 500,000 Da allow selective fractionation of proteins, pectins, and polyphenols, thereby eliminating turbidity-causing materials while simultaneously enriching target bioactive constituents in the retentate (Cassano et al., 2007; Girard & Fukumoto, 2000; Vaillant et al., 2001).

Beyond clarification, one of the major advantages of UF is its ability to preserve thermolabile compounds. The low-temperature operation of membrane processes therefore plays a crucial role in maintaining the structural integrity and biological activity of these molecules. Previous studies have reported that membrane filtration techniques can be effectively used to recover heat-sensitive natural compounds with high efficiency (Arend et al., 2017; Avram et al., 2017; Conidi et al., 2017). Collectively, this evidence highlights the growing relevance of membrane-based approaches for producing high-quality, functionally enriched fruit juice products. However, despite the widespread application of UF in other fruit matrices, studies specifically investigating the selective retention and concentration of betalains and phenolic compounds from red beetroot using membranes of different MWCO values remain limited. In their work Lee et al. (1982) demonstrated that sequential ultrafiltration and reverse osmosis could be applied to clarify beetroot juice and enhance betalain concentration after enzyme pretreatment. Similarly, nanofiltration using NF 200 membranes has been shown to effectively recover betalains and phenolic compounds from beetroot peel extracts, yielding enriched fractions with high pigment and antioxidant content (Zin and Bánvolgyi, 2021). These studies indicate the potential utility of membrane technologies for pigment recovery; however, comprehensive investigations explicitly comparing the effects of different MWCO membranes on red beetroot juice composition remain scarce. This underscores the need for systematic research aimed at understanding how membrane characteristics influence the separation, enrichment, and stability of beetroot bioactives during processing.

Therefore, the present study aimed to systematically evaluate the effect of ultrafiltration using membranes with distinct MWCO values (2 kDa and 5 kDa) on the chemical properties of red beetroot juice, followed by diafiltration experiments to further assess solute washout and selective purification. Diafiltration (DF) was applied after ultrafiltration to enhance the removal of low-molecular-weight solutes and to improve the purification of membrane-retained pigments and phenolic compounds. DF operates by adding fresh solvent to the retentate under constant-volume conditions, facilitating the washout of permeable solutes through dilution-driven mass transfer. This strategy has been shown to increase the purity of retentates, reduce matrix-associated fouling, and weaken concentration-polarization layers in various fruit juice systems (Conidi et al., 2020; Girard and Fukumoto, 2000). In this context, incorporating diafiltration following UF provides a clearer understanding of solute distribution dynamics and supports the selective enrichment of high-value phytochemicals while minimizing the presence of small co-solutes such as sugars and minerals.

Specifically, the study quantified the retention and concentration behaviors of betalains, phenolic compounds, sugars, and total soluble solids during both ultrafiltration and diafiltration, in addition to examining key membrane performance indicators such as permeate flux, fouling index, and cleaning efficiency. By addressing these knowledge gaps, this work provides deeper scientific insight into the membrane-based valorization of red beetroot juice and supports the development of efficient, non-thermal processing strategies for preserving and concentrating health-promoting phytochemicals.

## Material and methods

### Materials

All chemicals utilized in the analytical procedures were purchased from Merck (Darmstadt, Germany). The concentrate used for the preparation of beetroot juice was obtained from Meykon Fruit and Spring Water Industry and Trade Inc. (Antalya, Türkiye).

### Ultrafiltration and diafiltration of red beetroot juice

Ultrafiltration was performed using red beetroot juice (9.4° Brix), which was prepared by diluting a 68° Brix concentrate with demineralized water. Ultrafiltration (UF) experiments were carried out in a laboratory-scale ultrafiltration system (Sartorius, Göttingen, Germany) using Hydrosart filters (Sartorius AG, Germany) with molecular weight cut-offs of 2 and 5 kDa. The system consisted of a model 323 peristaltic pump (Watson-Marlow, Wilmington, MA) for circulation and two manometers for measuring inlet (P_in_) and outlet (P_out_) pressures. The inlet and outlet pressures were set to 2.5 and 0.5 bar, respectively. The feed was recirculated at constant pressure settings throughout all experiments, and the initial feed volume was 5 L. The filters used in the study had a membrane surface area of 1000 cm^2^ and were capable of withstanding pressures up to 4 bar. All experiments were carried out in batch mode at ambient laboratory temperature (25 °C ± 2), without the use of external temperature control systems. The process was continued until the volume reduction factor (VRF) of 10 was achieved. Permeate and retentate samples obtained from UF experiments were collected in amber bottles and stored at 4 °C until analysis.

Water permeability values of the membranes were determined as the slope of the water flux (J) versus transmembrane pressure (TMP) plot at a constant temperature (25 °C). Transmembrane pressure and flux values were calculated using Eqs. (1 and 2), respectively.

1 $$TMP=\frac{{P}_{in}+ {P}_{out}}{2}$$

In this equation, P_in_ represents the feed (inlet) pressure (bar), while P_out_ denotes the retentate (outlet) pressure (bar).

Water flux (J) was determined at constant TMP and temperature by measuring the permeate volume collected over a defined membrane surface area within a given time.

2 $$J=\frac{{V}_{p}}{A\times t}$$

Here, Vp is the permeate volume collected over time t through a membrane with surface area A.

Before the experiments, pure water flux (WP_0_) was measured at different TMP values at constant temperature (25 °C), and pure water permeability was calculated from the resulting graph. After ultrafiltration of red beetroot juice, pure water flux (WP_1_) was measured again. As part of the cleaning procedure, the membrane was rinsed with demineralized water for 30 min and cleaned with 1 N sodium hydroxide solution at 50 °C for 1 h. Subsequently, sodium hydroxide was removed with demineralized water, and pure water flux was measured once more (WP_2_). The fouling index (FI) was calculated using Eq. (3), comparing pure water permeabilities before and after juice filtration.

3 $$FI \left(\%\right)=[1-({WP}_{1}/{WP}_{0})]\times 100$$

Here, WP_1_ is the pure water permeability after red beetroot juice filtration, and WP_0_ is the pure water permeability of the unused membrane. Cleaning efficiency (CE) was calculated according to Eq. (4):

4 $$CE \left(\%\right)= {(WP}_{2}/{WP}_{0}) \times 100$$

Here, WP_2_ is the pure water permeability after chemical cleaning, and WP_0_ is the pure water permeability of the unused membrane.

The volume reduction factor (VRF) during ultrafiltration was calculated using Eq. (5):

5 $${\mathrm{VRF}}\, = \,{{{\mathrm{V}}_{{\mathrm{f}}} } \mathord{\left/ {\vphantom {{{\mathrm{V}}_{{\mathrm{f}}} } {\left( {{\mathrm{V}}_{{\mathrm{f}}} - {\mathrm{V}}_{{\mathrm{p}}} } \right)}}} \right. \kern-0pt} {\left( {{\mathrm{V}}_{{\mathrm{f}}} - {\mathrm{V}}_{{\mathrm{p}}} } \right)}}$$

where V_f_ and V_p_ represent the initial feed volume and the permeate volume, respectively.

The separation performance of a membrane can be expressed as the solute rejection ratio (R_j_). For the compounds analyzed in this study, the membrane rejection ratio was calculated using Eq. (6):

6 $${R}_{j }(\%)=(1-({C}_{p}/{C}_{f}))\times 100$$

where C_f_ and C_p_ denote the feed and permeate concentrations, respectively. R_j_ measures the proportion of solute retained by the membrane (Díaz-Reinoso et al., 2009).

In calculating the concentration factor (CF), which indicates the degree to which the compounds examined in this study were concentrated as a result of ultrafiltration, Eq. (7) was used.

7 $$CF=\frac{{C}_{r}}{{C}_{f}}$$

Here, C_r_ is the concentration of the compound in the retentate, and C_f_ is its concentration in the feed solution (Dushkova et al., 2022).

Diafiltration (DF) experiments were carried out using the selected membrane in batch mode following ultrafiltration. The retentate obtained after ultrafiltration was subjected to diafiltration in independent runs, each corresponding to a specific diafiltration volume (DV). For each experiment, deionized water was added to the retentate to reach a predetermined DV value (3, 6, or 9), after which the solution was filtered under the same operating conditions to obtain the corresponding retentate fractions (DV3-ret, DV6-ret, and DV9-ret). Thus, diafiltration experiments were performed separately for each DV level rather than as a continuous or sequential process. No continuous water addition was applied during diafiltration. In all diafiltration runs, the volume reduction factor (VRF) was maintained at 10. The experiments were conducted at 25 °C ± 2, with inlet and outlet pressures of 2.5 and 0.5 bar, respectively. The diafiltration volume (DV), defined as the ratio of the volume of water added to the retentate volume, was calculated according to Eq. (8):

8 $$DV= \frac{{V}_{w}}{{V}_{r}}$$

where V_w_ is the volume of water added during the diafiltration process and V_r_ is the volume of retentate.

Mass balance was calculated for each component by summing the mass recovered in the retentate and permeate fractions and comparing it to the initial mass in the feed. The mass balance (%) was expressed as:

9 $$Mass balance (\%)= \frac{{M}_{r}+ {M}_{p}}{{M}_{0}} \times 100$$

where M_r_ and M_p_ represent the mass of the component in the retentate and permeate, respectively, and M_0_ is the initial mass in the feed (Eq 9).

### Determination of total soluble solids (TSS) content

Soluble solids content (% Brix) was measured using a digital refractometer (Atago, Japan). Measurements were performed at 25 °C ± 0.5.

### Determination of total phenolic content (TPC)

Total phenolic content of the samples were determined according to the Folin–Ciocalteau’s method (Spanos and Wrolstad, 1990). Gallic acid was used as a calibration standard and results were expressed in unit of gallic acid equivalents (GAE) (mg GAE/L). Absorbance was measured by using a UV–visible spectrophotometer (Shimadzu UV-160A, Japan) at a wavelength of 765 nm.

### Determination of total betalains content (BC)

The total betalain content was determined using a spectrophotometer (Shimadzu UV-160A, Japan) following appropriate dilution. Absorbance was measured at 485 nm for betaxanthins and 536 nm for betacyanins, and the concentrations were calculated using the corresponding equation (Eq. 10 and 11) (Wruss et al., 2015).

10 $$BS \left(mg/L\right)=\frac{A\times MW\times DF\times 1000}{\varepsilon \times L}$$

11 Betalain=betaxanthin+betacyanin

where BS is betaxanthin or betacyanin; A is the absorbance; MW is the molecular weight (betaxanthin = 308 g/mol and betacyanin = 550 g/mol); DF is the dilution factor; ε is the molar extinction coefficient (betaxanthin = 48,000 L mol^−1^ cm^−1^ and betacyanin = 60,000 L mol^−1^ cm^−1^) and L is the path length (cm).

### Determination of sugar content

Sugar content was quantified using the 3,5-dinitrosalicylic acid (DNS) method. Absorbance was measured at 575 nm using a UV–Vis spectrophotometer (Shimadzu UV-160A, Japan). Quantification was performed using a sucrose calibration curve prepared at known concentrations, and deionized water was used as the blank (Roukas, 1994).

### Statistical analysis

All statistical analyses were performed using SAS software, Version 7 (SAS Institute Inc., Cary, NC). Ultrafiltration experiments were conducted in duplicate, and all analyses were performed in two parallels. Results for all evaluated parameters (n = 4) were presented as mean ± standard deviation and subjected to analysis of variance (ANOVA) at a significance level of 0.05.

## Results and discussion

### Membrane performance

Hydraulic permeabilities of the membranes before and after the cleaning procedure, as well as the fouling index (FI) and cleaning efficiency (CE), were calculated (Table 1). For the membrane with a 2 kDa MWCO, hydraulic permeability decreased by 16.30% following the ultrafiltration of red beetroot juice, whereas the decrease for the 5 kDa membrane was 15.01%.

**Table 1:** Water permeability (WP), fouling index (FI), and cleaning efficiency (CE) values of membranes in the ultrafiltration of red beetroot juice

	2 kDa	5 kDa
WP_0_ (L/m^2^hbar)	11.67	18.36
WP_1_ (L/m^2^hbar)	9.77	15.61
WP_2_ (L/m^2^hbar)	11.51	18.24
FI (%)	16.30	15.01
CE (%)	98.58	99.33

The negligible difference between the two MWCO values indicates that pore size had only a limited influence on fouling behavior under the experimental conditions. This finding is consistent with reports in the literature suggesting that fouling is often governed more by feed characteristics -such as suspended solids, polysaccharides, and phenolic compounds- than by MWCO alone (Cassano et al., 2011; Conidi et al., 2017; Yammine et al., 2019).

The high recovery of hydraulic permeability following the cleaning procedure, reaching approximately 99% of the initial values for both membranes, further suggests that fouling was predominantly reversible. The effectiveness of the cleaning protocol indicates that foulant deposition was likely associated with loosely attached cake layers or weakly adsorbed solutes rather than irreversible pore blockage. Overall, the similarity in fouling behavior between the two membranes, combined with their high permeability recovery, highlights that reversible fouling mechanisms were dominant and that the applied operational and cleaning conditions were well-suited for maintaining membrane performance in red beetroot juice ultrafiltration.

**Fig. 1:**
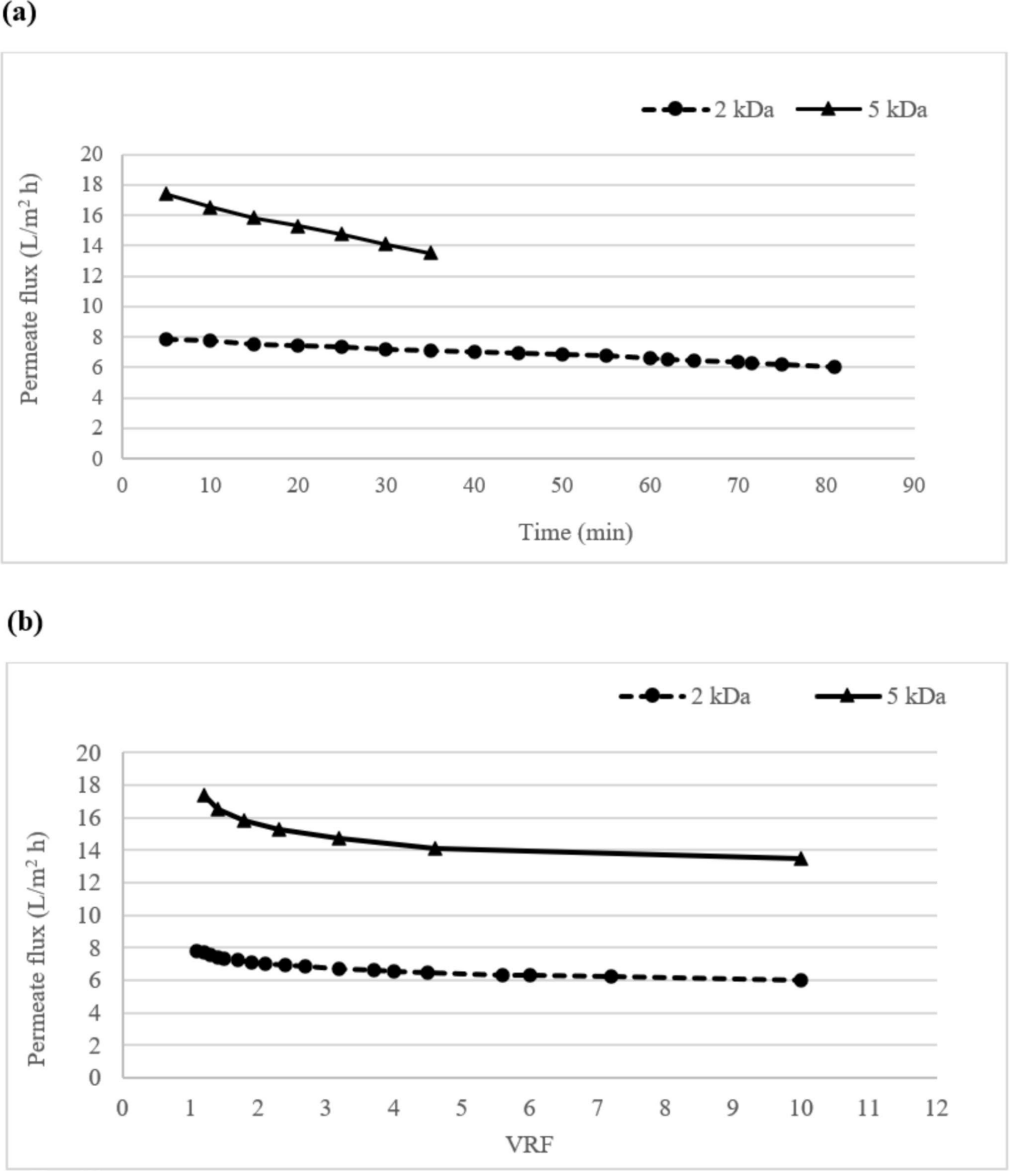
Variation of permeate flux as a function of time (**a**) and VRF (**b**)

The variation of permeate flux as a function of time is presented in Fig. 1a, while the relationship between permeate flux and the volume reduction factor (VRF) is shown in Fig. 1b. For the 2 kDa membrane, the initial permeate flux of 7.8 L/m^2^h decreased by 23% to 6 L/m^2^h at a VRF of 10. A similar decreasing trend was observed for the 5 kDa membrane, in which the initially higher permeate flux (17.4 L/m^2^h) declined by 22%, reaching 13.5 L/m^2^h. Although the 5 kDa membrane maintained a higher absolute flux throughout the process due to its larger pore size and lower intrinsic resistance, the proportional flux decline was comparable for both membranes. This suggests that, under the conditions tested, MWCO had a limited effect on mitigating concentration polarization or gel layer formation, phenomena widely recognized as dominant contributors to flux decline in fruit juice ultrafiltration (Cassano et al., 2018; Conidi et al., 2020). These findings agree with earlier studies showing that flux reduction is mainly driven by the accumulation of high-molecular-weight solutes and colloidal material at the membrane surface, increasing resistance with rising VRF (Koschuh et al., 2005). Although the 5 kDa membrane exhibited higher initial flux, its susceptibility to concentration polarization and gel layer formation was similar to that of the 2 kDa membrane, underscoring the dominant influence of feed composition and solute–membrane interactions on permeate flux behavior.

### Effect of ultrafiltration on the chemical properties of red beetroot juice

Table 2 summarizes the total phenolic content (TPC), betalain content (BC), total soluble solids (TSS), and sugar contents measured in the permeate and retentate fractions obtained using membranes with different MWCO values during ultrafiltration. For both membranes, the concentrations of these components in the permeate were consistently lower than those in the retentate, indicating selective retention of solutes within the membrane system. This observation is consistent with previous studies reporting that membrane processes effectively retain phenolic compounds and color constituents during fruit juice clarification, thereby preserving juice quality while separating high-molecular-weight components (Conidi et al., 2020). Among the operating conditions investigated, the 2 kDa membrane produced the most concentrated retentate, exhibiting the highest TPC (3352.23 mg GAE/L), BC (1206.59 mg/L), sugar content (128.17 g/L), and TSS (13.35%). The smaller pore size of the 2 kDa membrane likely enhanced solute retention by restricting the passage of phenolic compounds and pigments more effectively than the 5 kDa membrane. Similar effects of membrane cut-off on solute selectivity have been reported in the literature, where tighter membranes were associated with increased retention of desirable components and improved product enrichment (Conidi et al., 2017).

**Table 2:** Variation in total soluble solids (%), total phenolic content (mg GAE/L), total betalain content (mg/L), and sugar content (g/L) of red beetroot juice following ultrafiltration with 2 and 5 kDa membranes

Ultrafiltration		TSS (% Brix)	TPC (mg GAE/L)	BC (mg/L)	Sugar Content (g/L)
MWCO	Fraction				
Feed		9.40^c^ ± 0.01	1183.75^c^ ± 3.89	305.11^c^ ± 0.25	84.80^c^ ± 0.92
2 kDa	Permeate	8.65^d^ ± 0.05	704.82^e^ ± 0.72	120.79^e^ ± 0.08	79.76^d^ ± 0.56
	Retentate	13.35^a^ ± 0.05	3352.23^a^ ± 2.61	1206.59^a^ ± 2.41	128.17^a^ ± 3.75
5 kDa	Permeate	8.95^d^ ± 0.05	838.75^d^ ± 0.54	198.82^d^ ± 0.11	82.54^cd^ ± 0.62
	Retentate	11.10^b^ ± 0.01	2408.04^b^ ± 4.46	702.88^b^ ± 1.16	100.47^b^ ± 2.23

Statistically significant differences are indicated by different letters in the same column (P < 0.05)

Although membrane performance is often interpreted based on molecular weight cut-off (MWCO), the observed retention of phenolic compounds and betalains cannot be explained solely by size exclusion. The molecular weights of these compounds are generally lower than the nominal MWCO values of the membranes used, indicating that additional mechanisms contribute to their retention. In particular, solute–membrane interactions, such as adsorption and affinity effects, may play a significant role. Electrostatic interactions between charged solutes and the membrane surface can further influence transport behavior. Moreover, phenolic compounds and betalains may interact with other macromolecules present in the juice matrix, forming aggregates or complexes that increase their apparent molecular size. These combined mechanisms can enhance retention beyond that predicted by MWCO alone (Liu et al., 2018; Siebert et al., 1996).

Overall, these findings highlight the critical role of MWCO in determining solute selectivity, while also emphasizing that membrane performance is governed by a combination of size exclusion and physicochemical interactions. Consequently, membranes with lower MWCO values promote the formation of more concentrated retentate fractions during ultrafiltration of red beetroot juice.

### Concentration factor (CF)

**Fig. 2:**
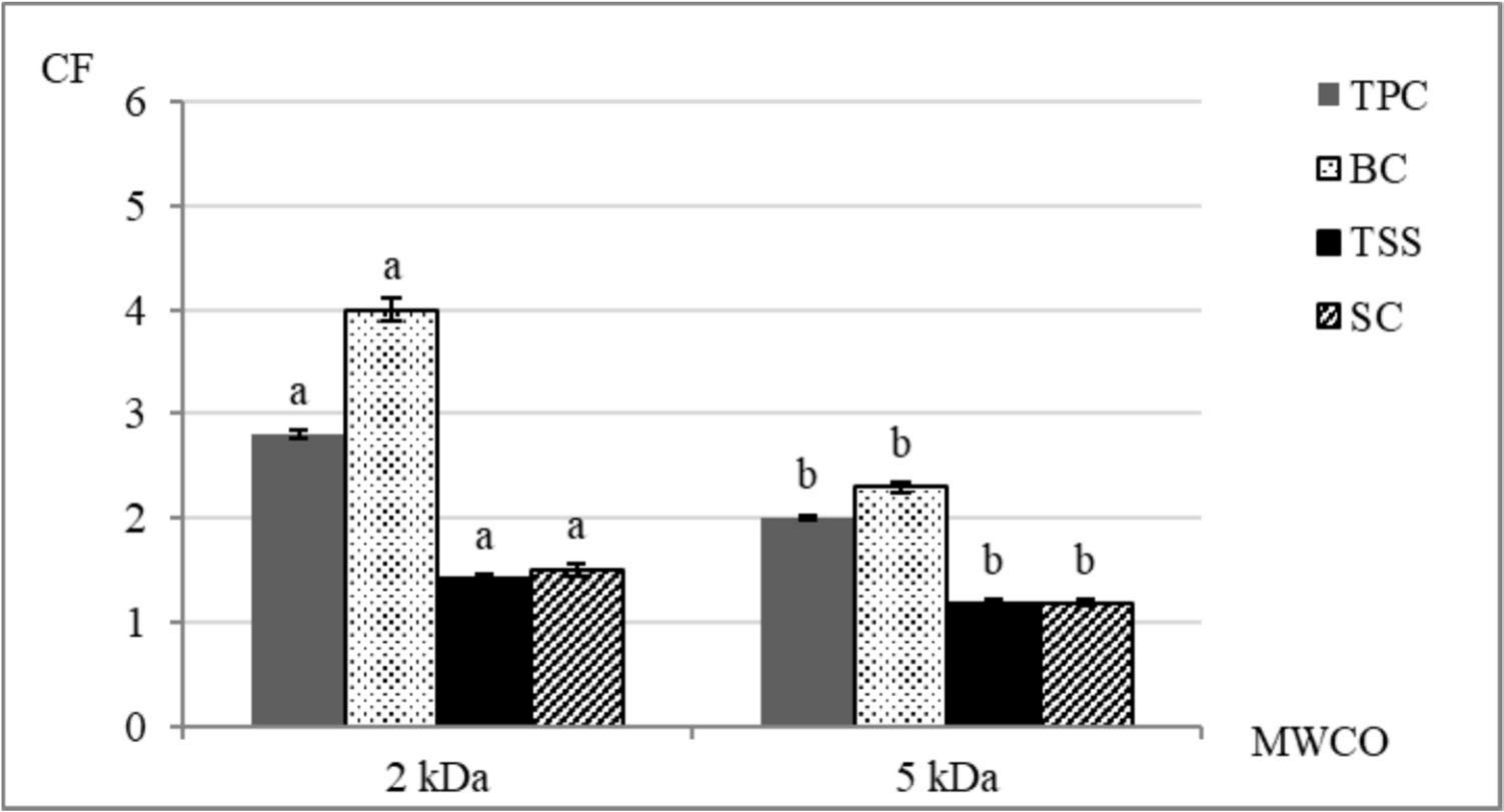
Effects of MWCO on the concentration factor (CF) of total phenolic content (TPC), total betalain content (BC), total soluble solids (TSS) and sugar content (SC) during ultrafiltration of red beetroot juice. Different letters indicate significant differences between 2 and 5 kDa membranes (P < 0.05)

The variation in concentration factors (CF) of the analyzed compounds as influenced by membrane MWCO during the ultrafiltration of red beetroot juice is presented in Fig. 2. As MWCO increased, the CF values for phenolic compounds decreased, yielding values of 2.8 and 2.0 for the 2 kDa and 5 kDa membranes, respectively. A similar trend was observed for betalains, whose CF values were markedly higher with the 2 kDa membrane (4.0) compared with the 5 kDa membrane (2.3).

In contrast, the concentration factors of total soluble solids and sugars remained substantially lower, with CF values of 1.42 and 1.51 for the 2 kDa membrane and 1.18 for both components when using the 5 kDa membrane. The limited concentration of these small molecular-weight solutes is expected, as their hydrophilic nature and low steric resistance allow them to permeate easily through both membranes. Overall, the higher CF values obtained with the 2 kDa membrane underscore its superior enrichment capability, indicating that ultrafiltration using lower MWCO membranes is highly effective for concentrating phenolic and betalain pigments while allowing selective removal of smaller solutes.

The concentration factor (CF) achieved during ultrafiltration is governed by the interplay between membrane rejection (R_j_) and the volume reduction factor (VRF), which together define the mass balance of solutes within the system. While R_j_ determines the fraction of solute retained versus permeated, VRF controls the extent of solvent removal and, consequently, the concentration of retained species.

From a mass balance perspective, CF increases with increasing VRF for solutes exhibiting high rejection, as their permeation is limited and accumulation in the retentate is favored. In contrast, for low-molecular-weight solutes with low rejection values, such as sugars and soluble solids, permeation dominates over retention, resulting in lower CF values despite volume reduction. Thus, CF reflects the dynamic equilibrium between convective transport toward the membrane, diffusive back-transport, and selective permeation through the membrane. This relationship highlights that effective solute enrichment during ultrafiltration requires both high membrane selectivity (R_j_) and sufficient volumetric concentration (VRF), as also reported in previous membrane filtration studies (Cassano et al., 2018; Díaz-Reinoso et al., 2009; Girard and Fukumoto, 2000).

### Rejection (membrane retention performance)

**Fig. 3:**
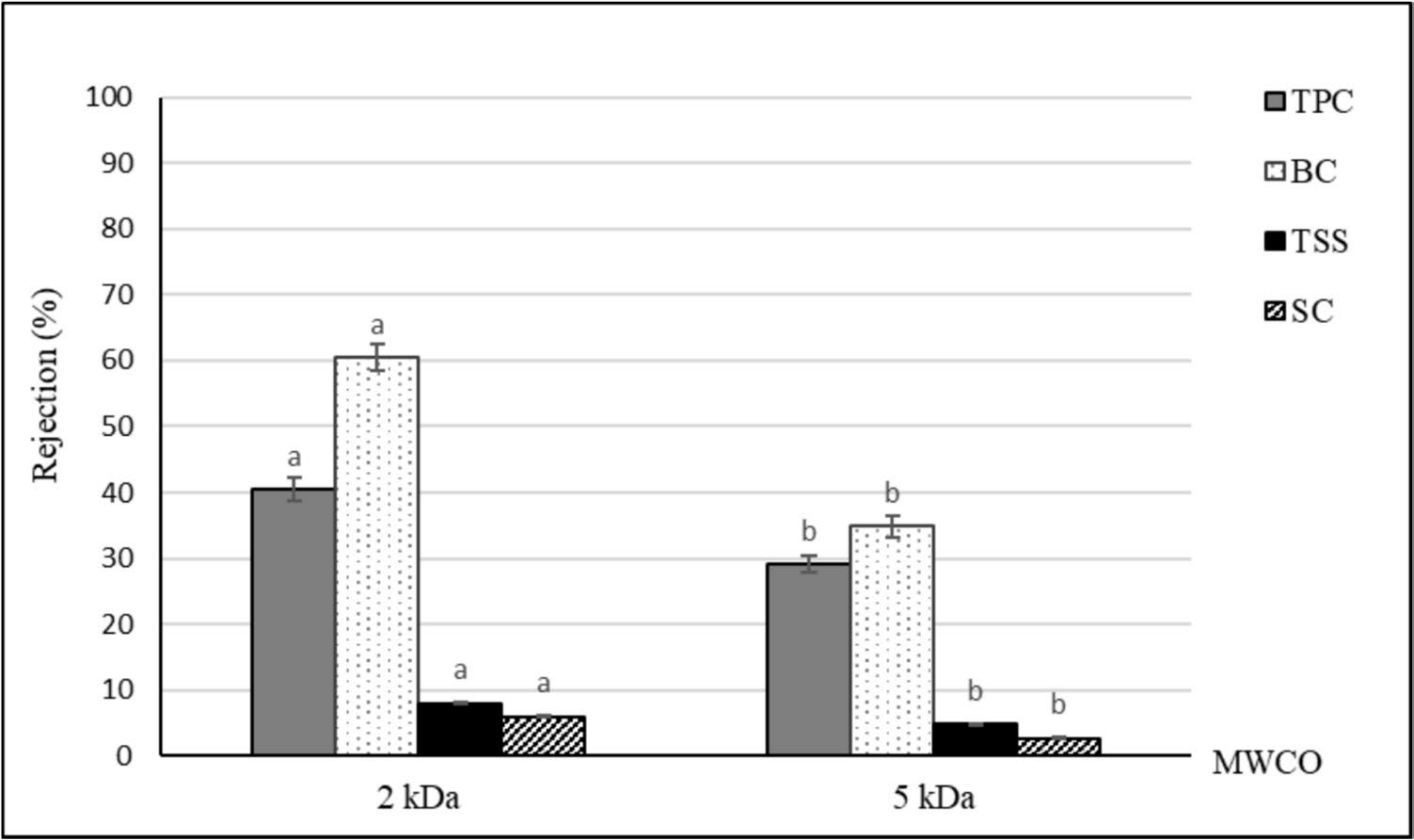
Effects of MWCO on the rejection ratio (R_j_) of total phenolic content (TPC), total betalain content (BC), total soluble solids (TSS) and sugar content (SC) during ultrafiltration of red beetroot juice. Different letters indicate significant differences between 2 and 5 kDa membranes (P < 0.05)

The rejection ratios (R_j_) of total phenolics, total betalains, sugars, and total soluble solids (TSS) in red beetroot juice concentrates obtained by ultrafiltration are shown in Fig. 3. A comparison of the membranes based on their molecular weight cut-off (MWCO) revealed distinct differences in solute rejection behavior. The 2 kDa membrane exhibited low rejection of total soluble solids (TSS) and sugars (7.98% and 5.94%, respectively), whereas substantially higher rejection values were observed for phenolic compounds and betalains (40.46% and 60.41%, respectively), indicating preferential retention of these bioactive constituents. In contrast, the use of the 5 kDa membrane, characterized by a larger pore size, resulted in reduced rejection across all components, with TSS (4.79%), sugars (2.67%), total phenolic compounds (29.14%), and betalains (34.84%) exhibiting lower retention compared to the 2 kDa membrane. These findings demonstrate that ultrafiltration performed with the 2 kDa membrane provides enhanced selectivity toward phenolic compounds and betalains, making it more effective for the enrichment of these bioactive components. Therefore, diafiltration studies were performed by using 2 kDa membrane filter as described in the relevant section.

Membrane technologies such as ultrafiltration and nanofiltration have been explored in the literature for the recovery and concentration of betalains, phenolic compounds, and other bioactive constituents from beetroot and beetroot-derived matrices. For instance, nanofiltration of beetroot peel extracts using an NF 200 membrane at 40 bar and 30 °C produced permeate fractions containing substantial amounts of betaxanthins (202.25–206.62 mg/L) and betacyanins (360.07–339.72 mg/L), alongside high phenolic content (987.79–972.72 mg/L) and antioxidant activity (642.06–745.97 mg/L), demonstrating that membrane processes can retain and even concentrate color and phenolic compounds compared to crude extracts (Zin and Bánvölgyi, 2021). Moreover, previous studies on membrane concentration have indicated that ultrafiltration and reverse osmosis can yield highly purified and concentrated betalain fractions, highlighting the feasibility of these techniques for valorizing betalain-rich juices and extracts (Lee et al., 1982). Pressure-driven membrane coupling has also been investigated for selective separation of betacyanins (Tamba et al., 2019). These findings corroborate our observation that lower MWCO membranes (e.g., 2 kDa) are effective at retaining betalains and phenolics during red beetroot juice ultrafiltration, supporting the use of selective membrane separation to enhance the concentration of valuable bioactive compounds in the retentate.

Previous studies have reported that membrane rejection behavior is governed not only by steric hindrance but also by a range of physicochemical interactions such as solute-membrane affinity, electrostatic interactions, and interactions among different chemical groups present in fruit juices, which may promote the formation of macromolecular aggregates near or within the membrane structure (Siebert et al., 1996). Such aggregation and interaction phenomena can modify the effective molecular size of pigments, phenolics, and polysaccharides, thereby altering their transport and retention during ultrafiltration. Consistent with these observations, the mass balance evaluation in UF systems frequently reveals solute–membrane interactions and the subsequent adsorption of solutes onto the membrane surface or within the pores as major contributors to material losses (Liu et al., 2018).

### Diafiltration of red beetroot juice

Diafiltration was applied to enhance the removal of low-molecular-weight sugars while preserving high-value phenolic compounds and betalain pigments retained by the membrane. The performance of the 2 kDa membrane was evaluated by integrating diafiltration up to a diafiltration volume (DV) of 9. In the batch diafiltration experiments performed with the 2 kDa membrane, both total phenolic compounds and betalains exhibited a progressive decrease in concentration with increasing diafiltration volume. Specifically, TPC values declined from 4941.07 to 3298.21 and 2189.29 mg GAE/L in DV3-ret, DV6-ret, and DV9-ret, respectively, while total betalain concentrations decreased from 1872.70 to 1265.32 and 791.54 mg/L. This trend is primarily attributed to dilution effects associated with diafiltration. When the effect of diafiltration on total soluble solids (TSS) and sugar content was considered, the TSS values were 5.5%, 3.7%, and 4.65% in DV3-ret, DV6-ret, and DV9-ret, respectively, while the corresponding sugar contents were 52.1, 34.51, and 42.68 g/L. Although increasing diafiltration volume generally facilitates the removal of low-molecular-weight solutes such as sugars, the non-monotonic trend observed at higher DV may be attributed to mass transfer limitations and fouling effects under varying concentration conditions. The recovery rates in the diafiltration retentates remained comparatively high, ranging from 58.96 to 68.87% for TPC and from 62.1 to 73.4% for betalains. The highest recovery values for both TPC and BC were observed at DV 6, reaching 68.87% and 73.4%, respectively. At this condition, 17.62% of the total soluble solids (TSS) and 17.86% of the sugar content were retained in the DV 6 retentate.

**Fig. 4:**
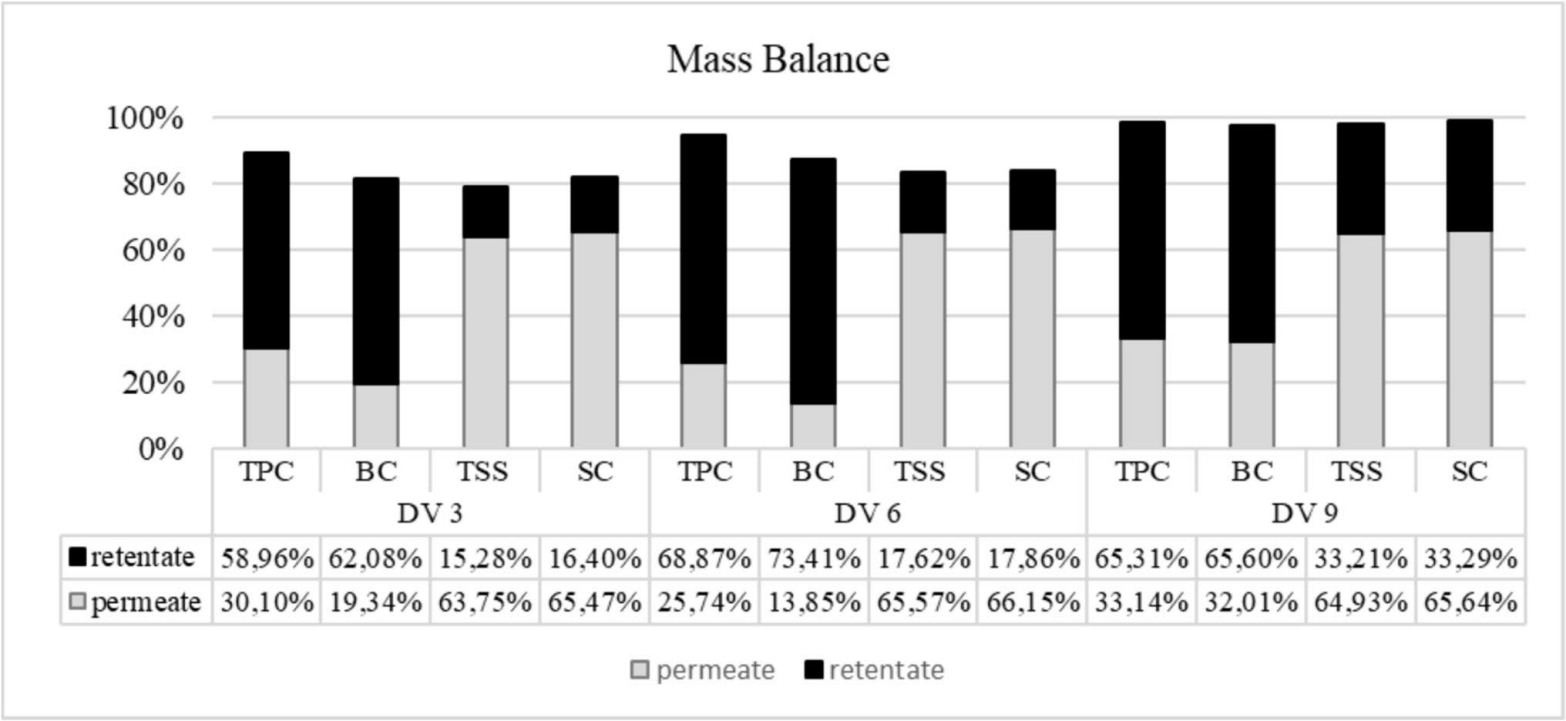
Mass balance of total phenolic content (TPC), total betalain content (BC), total soluble solids (TSS), and sugar content (SC) in retentate and permeate fractions at different diafiltration volumes (DV)

The mass balance of total phenolic content (TPC), total betalain content (BC), total soluble solids (TSS), and sugar content (SC) in the retentate and permeate fractions was analyzed as a function of diafiltration volume (DV), as shown in Fig. 4, providing further insight into solute distribution and overall process performance. The mass of each component in the retentate and permeate fractions was compared to its initial mass in the feed. The mass balance (%) was determined as the ratio of the total recovered mass to the initial mass of the component, expressed as a percentage.

At DV 9, the mass balance for all components approached 100%, indicating minimal solute loss and suggesting that the system operated close to ideal conditions. This behavior can be attributed to the dilution effect, which reduces solution viscosity and mitigates fouling and concentration polarization phenomena, thereby enhancing mass transfer. In contrast, noticeable deviations from complete mass balance were observed at lower diafiltration volumes (DV 3 and DV 6), where partial losses of solutes occurred. Despite similar amounts of sugars being transferred to the permeate across all DV conditions, the reduced overall recovery at lower DV suggests that a fraction of the solutes remained retained within the membrane system. This behavior is likely associated with increased fouling and the formation of a cake layer under more concentrated conditions, which can impose additional resistance to mass transfer and promote the retention of even low-molecular-weight compounds such as sugars. These findings indicate that, in addition to membrane selectivity, hydrodynamic conditions and fouling behavior play a critical role in determining solute recovery and overall process efficiency during diafiltration.

Collectively, DV 6 offers an optimal balance between bioactive retention and sugar removal, consistent with previous studies demonstrating that diafiltration selectively removes low-molecular-weight solutes while preserving phenolic compounds (Almanasrah et al., 2015; Conidi et al., 2022).

The results obtained in this study demonstrate the potential applicability of the integrated UF–DF process for the valorization of red beetroot juice in industrial settings. The selective enrichment of phenolic compounds and betalains, combined with the removal of sugars, provides a promising approach for the production of functional ingredients with enhanced bioactive content. In particular, the retentate fraction obtained through this process could be utilized as a natural colorant or antioxidant-rich ingredient in food formulations, while the sugar-enriched permeate may find applications in fermentation processes or as a source of natural sugars. Furthermore, the use of membrane-based technologies offers significant advantages over conventional thermal processes, including lower energy consumption, mild operating conditions, and improved preservation of heat-sensitive compounds such as betalains.

These features highlight the potential of UF–DF processes as scalable and sustainable alternatives for the production of high-value products from plant-based matrices. Future work may focus on optimizing diafiltration parameters, scaling the process, and evaluating the stability and functional properties of the recovered fractions.

## Data Availability

The data presented in this study are available on request from the corresponding author.
